# Associations of preoperative pan-immune inflammation value with postoperative acute kidney injury following open-heart surgery: a retrospective analysis

**DOI:** 10.3389/fcvm.2026.1920153

**Published:** 2026-09-24

**Authors:** Yingling Zhong, Pingxiong Zeng, Weichang Wang, Jingdan Deng, Zhi Chen

**Affiliations:** Department of Anesthesiology, Meizhou People’s Hospital (Meizhou Academy of Medical Sciences), Meizhou, Guangdong, China

**Keywords:** acute kidney injury, cardiac surgery, cardiopulmonary bypass, pan-immune inflammation value, risk stratification

## Abstract

**Background:**

Cardiac surgery-associated acute kidney injury (CSA-AKI) is prevalent and results in significant morbidity. The pan-immune inflammation value (PIV), derived from routine blood counts, captures innate, adaptive, and coagulation-inflammation pathways; however, its preoperative association with CSA-AKI remains unclear.

**Methods:**

This retrospective cohort study encompassed 725 patients undergoing open-chest cardiac surgery with cardiopulmonary bypass. CSA-AKI was defined according to KDIGO criteria. The relationship between PIV and CSA-AKI was evaluated using multivariable logistic regression. A nonlinear relationship was investigated through restricted cubic spline (RCS) analysis. Subsequent subgroup analysis and interaction effect tests were conducted. The incremental predictive value of PIV was assessed using integrated discrimination improvement (IDI) and net reclassification improvement (NRI).

**Results:**

CSA-AKI was identified in 256 (35.3%) patients. Elevated preoperative PIV exhibited a dose-dependent association with increased CSA-AKI incidence (adjusted odds ratio per 50-unit increase: 1.04; 95% confidence interval: 1.02–1.06; *p* = 0.005). The preoperative PIV demonstrated a monotonic yet attenuating association with CSA-AKI incidence (*p*-non-linear = 0.087). Results were consistent across subgroups, with no significant interaction effect detected. PIV demonstrated a statistically significant yet clinically modest improvement in IDI and continuous NRI.

**Conclusion:**

An elevated preoperative PIV correlates with a heightened risk of CSA-AKI. Integrating PIV into preoperative risk algorithms may enhance risk stratification and facilitate targeted preventive measures.

## Introduction

Cardiac surgery-associated AKI (CSA-AKI) is a prevalent and serious adverse event, occurring in approximately one-third to one-half of patients undergoing such procedures (1–5). Among these individuals, an estimated 2%–3% progress to a stage requiring dialysis (6–8). CSA-AKI substantially increases mortality rates (9–11) and is closely associated with subsequent risks of chronic kidney disease (CKD) (12), end-stage kidney disease (13), cardiac dysfunction (14), and major cardiovascular complications (15). Therefore, early risk stratification and intervention are essential for optimizing patient recovery. The pathogenesis of CSA-AKI involves hemodynamic disturbances, inflammation, oxidative stress, and direct tubular injury (16). Cardiopulmonary bypass (CPB) and surgical trauma trigger a systemic inflammatory response, significantly contributing to CSA-AKI by promoting renal hypoperfusion and tubular damage (17).

Traditional renal markers, such as serum creatinine (Scr) and urine output, often remain unchanged until considerable renal injury has occurred, leading to diagnostic delays and missed opportunities for early intervention (18). Recently, composite inflammatory indices, including the C-reactive protein/albumin ratio, neutrophil-to-lymphocyte ratio (NLR), systemic immune-inflammation index (SII), monocyte-to-lymphocyte ratio (MLR), and monocyte × platelet/lymphocyte ratio (MLPR), have been employed to predict CSA-AKI. These indices may predict CSA-AKI either preoperatively or postoperatively (19–21). However, their reliance on only two or three blood-cell counts limits their ability to simultaneously capture the interplay between innate immunity, adaptive immunity, and the coagulation-inflammation axis. The novel pan-immune inflammation value (PIV) is calculated as (neutrophil count × platelet count × monocyte count) divided by lymphocyte count (22). Neutrophils and monocytes are critical components of the innate immune system and actively participate in the inflammatory cascade triggered by cardiac surgery (23). Platelets enhance inflammation through leukocyte interactions and the release of pro-inflammatory mediators (24), while lymphocytes regulate the immune response and help control excessive inflammation (25). Thus, PIV theoretically provides a more comprehensive assessment of the global immune-inflammatory-coagulation milieu.

In patients undergoing aortic dissection repair, elevated preoperative PIV correlates with increased mortality and postoperative complications (26). PIV, therefore, shows promise for early and accurate prediction of AKI, facilitating perioperative risk stratification and timely intervention. However, the relationship between PIV and CSA-AKI within the broader cardiac surgery population remains to be clearly established. This research aims to investigate the association between preoperative PIV and CSA-AKI, assessing its prognostic value and addressing the gap in utilizing PIV to identify high-risk individuals prior to surgery. It is hypothesized that elevated preoperative PIV serves as a significant and independent predictor of increased CSA-AKI incidence.

## Methods and materials

### Ethics statement and trial registration

This retrospective cohort study was conducted at Meizhou People's Hospital in accordance with the ethical principles outlined in the Declaration of Helsinki. Approval for the study protocol was granted by the Meizhou People's Hospital Ethics Committee (Approval No. 2025-C-122) and registered in the Chinese National Medical Research Registry (No. MR-44-25-057875). Due to the study's retrospective design and the use of anonymized data, ethical approval was obtained without the requirement for individual informed consent.

### Study population

The Clinical Data Center at Meizhou People's Hospital, encompassing data from January 2019 to February 2025, served as the source for the study cohort. Inclusion criteria consisted of: (1) adult patients (≥18 years); (2) patients who underwent open-heart surgery with CPB. Patients were excluded based on the following criteria: (1) lack of preoperative neutrophil, lymphocyte, platelet, and monocyte counts; (2) missing Scr measurements or incomplete medical records within 7 days postoperatively.

### Data collection

Demographic characteristics and comorbidities included age, gender, body mass index (BMI), hypertension, diabetes, and ischemic heart disease (IHD). Laboratory parameters measured encompassed albumin, the triglyceride-glucose (TyG) index, Scr, blood urea nitrogen (BUN), and hemoglobin. Recorded preoperative medications included antihypertensives, lipid-lowering agents, insulin, oral hypoglycemics, and diuretics. Additionally, preoperative left ventricular ejection fraction (LVEF) and coronary angiography information were collected. Surgical variables comprised American Society of Anesthesiologists (ASA) class, duration of anesthesia, CPB duration, aortic cross-clamp (ACC) duration, time-weighted area under the curve (TW-AUC) for mean arterial pressure (MAP) <60 mmHg, intraoperative blood loss, urine output, crystalloid and colloid volumes, red blood cell and plasma transfusions, and types of surgery.

### PIV and other indices calculation

The PIV was calculated using the formula: (neutrophil count × platelet count × monocyte count)/lymphocyte count (all in units of 10^9^). The formulas for calculating other indices are detailed in Supplementary Table S1.

### CSA-AKI definition

CSA-AKI was identified based on Kidney Disease: Improving Global Outcomes (KDIGO) guidelines: (a) an increase in Scr of at least 26.5 μmol/L within 48 h, or (b) an increase in Scr to 1.5 times or more above baseline levels within a seven-day period (27).

### Statistical analysis

Variables with <20% missing data were imputed using random-forest multiple imputation (Supplementary Table S2). Non-normally distributed continuous variables are presented as median (interquartile range, IQR) and compared using the Kruskal–Wallis or Wilcoxon rank-sum tests. Categorical variables are expressed as counts (percentages) and compared using Pearson's *χ*^2^ test. Receiver-operating characteristic (ROC) curves were utilized to assess the predictive performance of PIV, NLR, SII, MLR, and MLPR for CSA-AKI, and the area under the curves (AUCs) were compared using DeLong's test. Multivariable logistic regression analyzed the association between PIV and CSA-AKI, reporting odds ratios (ORs) with 95% confidence intervals (CIs). PIV was evaluated both as a continuous variable to characterize its dose-response relationship with CSA-AKI and in quartiles to refine risk stratification, facilitating intuitive comparisons across risk levels. Information on multicollinearity among baseline variables is provided in Supplementary Table S3. Variables with *p* < 0.05 in univariable analyses (detailed in Table 1) were incorporated into the final model. Restricted cubic spline (RCS) analysis examined potential nonlinear relationships between PIV and CSA-AKI risk in the entire cohort. Subgroup analyses were performed to assess whether the association between preoperative PIV and CSA-AKI was consistent across patient subgroups and to identify potential effect modification by clinical characteristics. To enhance covariate balance, a sensitivity analysis employing propensity score matching (PSM) was performed to calibrate baseline characteristics between the high- and low-preoperative PIV groups. To evaluate whether PIV could enhance patient risk stratification in clinical practice, its clinical utility was quantified through integrated discrimination improvement (IDI), net reclassification improvement (NRI) analyses and decision curve analysis (DCA), reinforcing PIV's independent predictive value. A mediation analysis model was constructed to systematically evaluate the mediating roles of intraoperative variables, elucidating the mechanistic pathway by which preoperative PIV contributes to CSA-AKI. The robustness of the PIV-CSA-AKI association was confirmed by assessing the attenuation of the PIV effect size after adjustment for these operative variables.

**Table 1:** Baseline characteristics stratified by the CSA-AKI and Non-CSA-AKI.

Characteristic	Overall	Non-CSA-AKI	CSA-AKI	*p*
	*N* = 725	*N* = 469	*N* = 256	
Preoperative PIV	243 (150, 427)	207 (137, 365)	308 (195, 606)	<0.001
Age (years)	58 (50, 65)	57 (49, 64)	60 (53, 66)	0.004
Gender (woman)	367 (50.6)	269 (57.4)	98 (38.3)	<0.001
BMI (kg/m^2^)	22.5 (20.0, 24.8)	21.9 (19.6, 24.2)	23.5 (21.3, 25.9)	<0.001
Preoperative medical histories				
Hypertension	210 (29.0)	107 (22.8)	103 (40.2)	<0.001
Diabetes	100 (13.8)	48 (10.2)	52 (20.3)	<0.001
Ischemic heart diseases	376 (51.9)	228 (48.6)	148 (57.8)	0.018
Preoperative laboratory results				
Albumin (g/L)	39.0 (36.6, 41.6)	39.5 (36.9, 42.0)	38.1 (36.4, 40.6)	<0.001
TyG index	7.15 (6.82, 7.53)	7.11 (6.79, 7.46)	7.27 (6.95, 7.67)	<0.001
Serum creatinine (umol/L)	82 (70, 101)	81 (67, 98)	84 (73, 104)	0.006
BUN (mmol/L)	6.08 (4.71, 7.89)	5.85 (4.58, 7.84)	6.55 (5.00, 7.99)	0.042
Uric acid (umol/L)	411 (335, 504)	399 (334, 491)	440 (335, 524)	0.006
Hemoglobin (g/L)	135 (123, 148)	133 (123, 147)	138 (123, 148)	0.202
Preoperative medications				
Antihypertensive medications	506 (69.8)	302 (64.4)	204 (79.7)	<0.001
Lipid-lowering medications	272 (37.5)	150 (32.0)	122 (47.7)	<0.001
Anti-diabetic medications	84 (11.6)	45 (9.6)	39 (15.2)	0.023
Diuretic medications	672 (92.7)	434 (92.5)	238 (93.0)	0.831
Preoperative LVEF (%)	63 (57, 68)	63 (58, 68)	62 (56, 68)	0.145
ASA classifications				<0.001
II–III	215 (29.7)	163 (34.8)	52 (20.3)	
IV–V	510 (70.3)	306 (65.2)	204 (79.7)	
Surgical types				<0.001
Great vessels	57 (7.9)	27 (5.8)	30 (11.7)	
Valves	508 (70.1)	324 (69.1)	184 (71.9)	
CABG and valves	28 (3.9)	15 (3.2)	13 (5.1)	
CABG	45 (6.2)	27 (5.8)	18 (7.0)	
Other surgeries	87 (12.0)	76 (16.2)	11 (4.3)	

The non-normally distributed continuous variables (determined by the Shapiro–Wilk test) are presented as median (interquartile range, 25%, 75%). The categorical variables are expressed as counts (percentage). CSA-AKI: cardiac surgery associated-acute kidney injury; PIV: pan-immune inflammation value; BMI, body mass index; TyG, triglyceride-glucose index; TyG = ln[triglyceride(mg/dL) × fasting blood glucose (mg/dL)/2]; BUN, blood urea nitrogen; LVEF, left ventricular ejection fraction; ASA classifications, American society of anesthesiologists; CABG, coronary artery bypass grafting. Other surgery types included congenital heart disease surgery and cardiac myxoma surgery.

All analyses were performed using R Foundation software (version 4.2, Vienna, Austria), with a significance level of *p* < 0.05 applied for two-sided tests.

## Results

### Baseline information

Seven hundred twenty-five patients undergoing open-heart surgery with CPB were identified and stratified into CSA-AKI (*n* = 256) and non-CSA-AKI (*n* = 469) groups (Figure 1). No patient in this cohort underwent unplanned reoperation. Patients with CSA-AKI exhibited distinct preoperative and intraoperative parameters compared to those without CSA-AKI (Table 1). Specifically, individuals with CSA-AKI had a higher preoperative PIV (308 vs. 207, *p* < 0.001), greater age (60 vs. 57 years, *p* = 0.004), elevated BMI (23.5 vs. 21.9 kg/m^2^, *p* < 0.001), and a higher TyG index (7.27 vs. 7.11, *p* < 0.001). Additionally, the rates of preoperative hypertension (40.2% vs. 22.8%, *p* < 0.001), diabetes (20.3% vs. 10.2%, *p* < 0.001), and IHD (57.8% vs. 48.6%, *p* = 0.018) were significantly higher in the CSA-AKI group. Supplementary Table S4 details the baseline characteristics stratified by preoperative PIV quartiles. The incidence of CSA-AKI significantly increased across PIV quartiles, ranging from 20.4% in the first quartile to 51.7% in the fourth quartile (*p* < 0.001).

**Figure 1:**
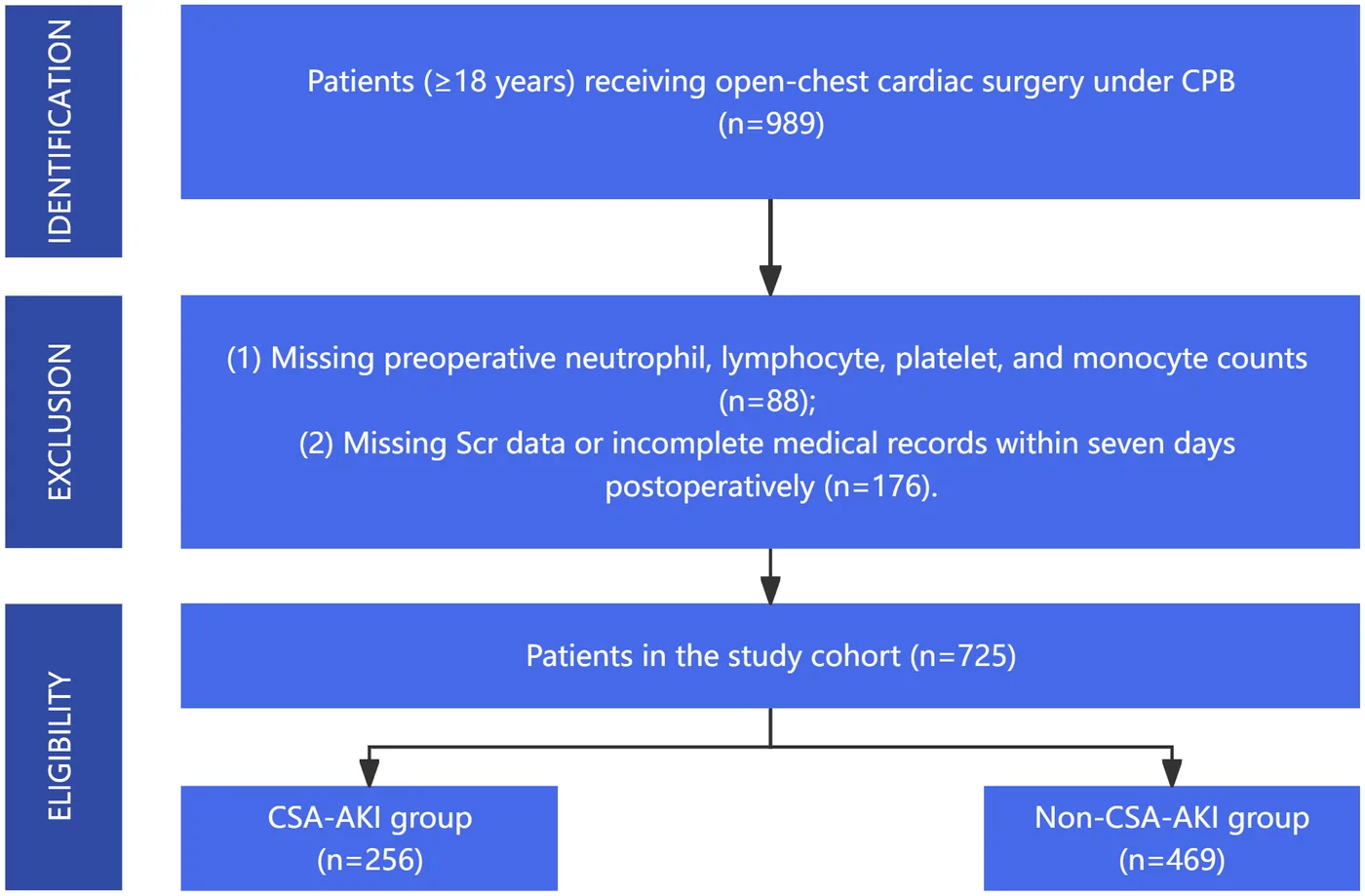
Flowchart of patient selection. The flowchart illustrates the patient selection process. The final cohort comprised 725 patients, including 469 without CSA-AKI and 256 with CSA-AKI. AKI, acute kidney injury; CSA-AKI, cardiac surgery-associated acute kidney injury.

### The results of ROC analysis, logistic regression and RCS analysis

PIV demonstrated the highest predictive accuracy among preoperative inflammatory biomarkers (Supplementary Table S5), with an AUC of 0.641 (95% CI: 0.599–0.683), outperforming SII, MLPR, NLR, MLR, and platelet-to-lymphocyte ratio (PLR). DeLong's test revealed that PIV had a statistically better discriminative performance compared to all other evaluated biomarkers (all *p* < 0.001). The optimal preoperative PIV cutoff was established at 231, yielding a sensitivity of 68.8% and a specificity of 55.7%.

Table 2 summarizes the logistic regression analysis investigating the relationship between preoperative PIV and CSA-AKI. In Model 3, each 50-unit increase in continuous PIV correlated with a 4.0% increase in CSA-AKI odds (OR: 1.04, 95% CI: 1.02–1.06, *p* = 0.005). When stratified by PIV quartiles, the fourth quartile (Q4, ≥427) displayed a significantly elevated risk compared to the lowest quartile (Q1, <150) (OR: 3.23, 95% CI: 1.90–5.48, *p* < 0.001). A significant dose-response relationship across PIV quartiles was noted, with CSA-AKI incidence rising from 20.4% in Q1 to 51.7% in Q4. Increasing preoperative PIV levels exhibited a monotonic but attenuating association with progressively elevated CSA-AKI risk (*p*-overall <0.001, *p*-non-linear = 0.087) (Figure 2).

**Table 2:** Multivariable logistic regression analysis of preoperative PIV and CSA-AKI risk.

Variables	Events (%)	Model 1		Model 2		Model 3	
		OR (95% CI)	*p*	OR (95% CI)	*p*	OR (95% CI)	*p*
Continuous PIV per 50 units	256 (35.3)	1.04 (1.03–1.06)	<0.001	1.04 (1.02–1.06)	<0.001	1.04 (1.02–1.06)	0.005
PIV quartile groups							
Q1 (<150)	37 (20.4)	Reference		Reference		Reference	
Q2 (150–242)	53 (29.3)	1.61 (0.99–2.61)	0.053	1.32 (0.79–2.21)	0.291	1.31 (0.77–2.20)	0.320
Q3 (243–426)	72 (39.8)	2.57 (1.61–4.11)	<0.001	2.01 (1.21–3.33)	0.007	2.08 (1.24–3.49)	0.006
Q4 (≥427)	94 (51.7)	4.16 (2.62–6.61)	<0.001	3.37 (2.02–5.61)	<0.001	3.23 (1.90–5.48)	<0.001

Model 1: crude. Model 2: adjust for: age, gender, BMI, hypertension, diabetes, ischemic heart disease, preoperative albumin, TyG index, creatinine, BUN, uric acid, antihypertensive agents, lipid-lowering drugs, and anti-diabetic drugs. Model 3: Adjust for: adjusted variables in the Model 2, ASA class, and surgical category. OR, odds ratio; CI, confidence interval; CSA-AKI, cardiac surgery associated-acute kidney injury; PIV, pan-immune inflammation value; BMI, body mass index; TyG, triglyceride-glucose index; BUN, blood urea nitrogen; ASA classifications, American society of anesthesiologists.

**Figure 2:**
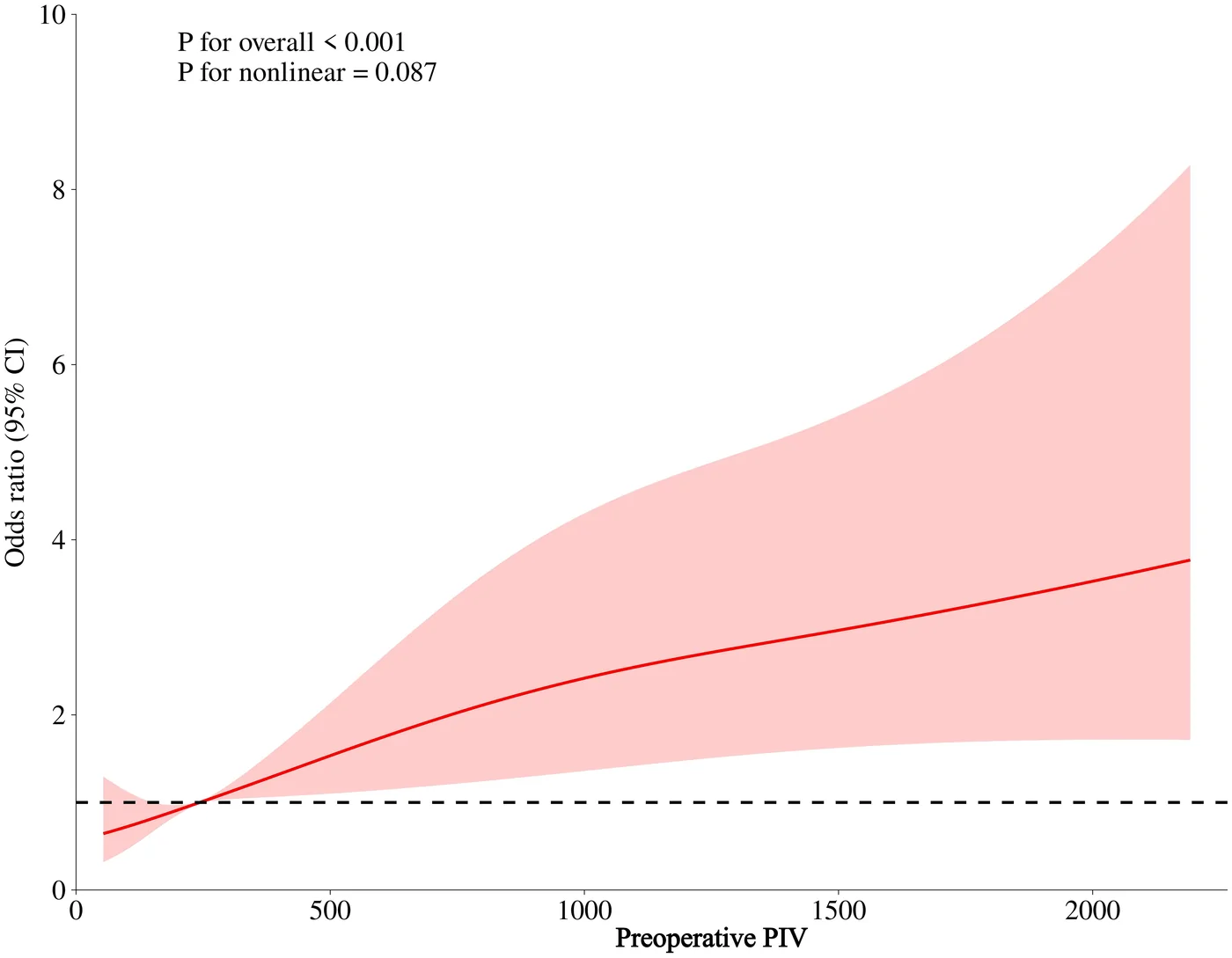
Restricted cubic spline analysis of the relationship between preoperative PIV and CSA-AKI risk. The curve was adjusted for all covariates listed in Table 2, Model 3. The *y*-axis represents the adjusted odds ratio (OR) for CSA-AKI, and the *x*-axis represents the preoperative pan-immune inflammation value (PIV). The solid line indicates the adjusted OR, and the shaded band indicates the 95% confidence interval. The model was fitted using restricted cubic splines with 4 knots.

### Post-hoc subgroup analysis and sensitivity analysis

A preoperative PIV ≥ 231 remained independently associated with increased CSA-AKI risk (overall OR 2.33, 95% CI 1.64–3.33, *p* < 0.001), with this association consistent across all subgroups (Figure 3). No statistically significant subgroup interaction effects were observed. A PSM sensitivity analysis was also performed to address the substantial differences in baseline characteristics between CSA-AKI and non-CSA-AKI groups. Using a PIV cutoff of 231, the cohort was divided into a high-PIV group (≥231) and a low-PIV group (<231), with 1:1 PSM conducted using surgical type as the matching variable. After matching, 268 patients were retained in each group, with baseline characteristics well balanced between both groups (Supplementary Table S6). In the multivariable logistic regression analysis of the PSM-matched cohort (Supplementary Table S7), the high-PIV group (≥231) exhibited a significantly higher risk of CSA-AKI compared to the low-PIV group (<231) (OR = 1.92, 95% CI 1.33–2.76, *p* < 0.001).

**Figure 3:**
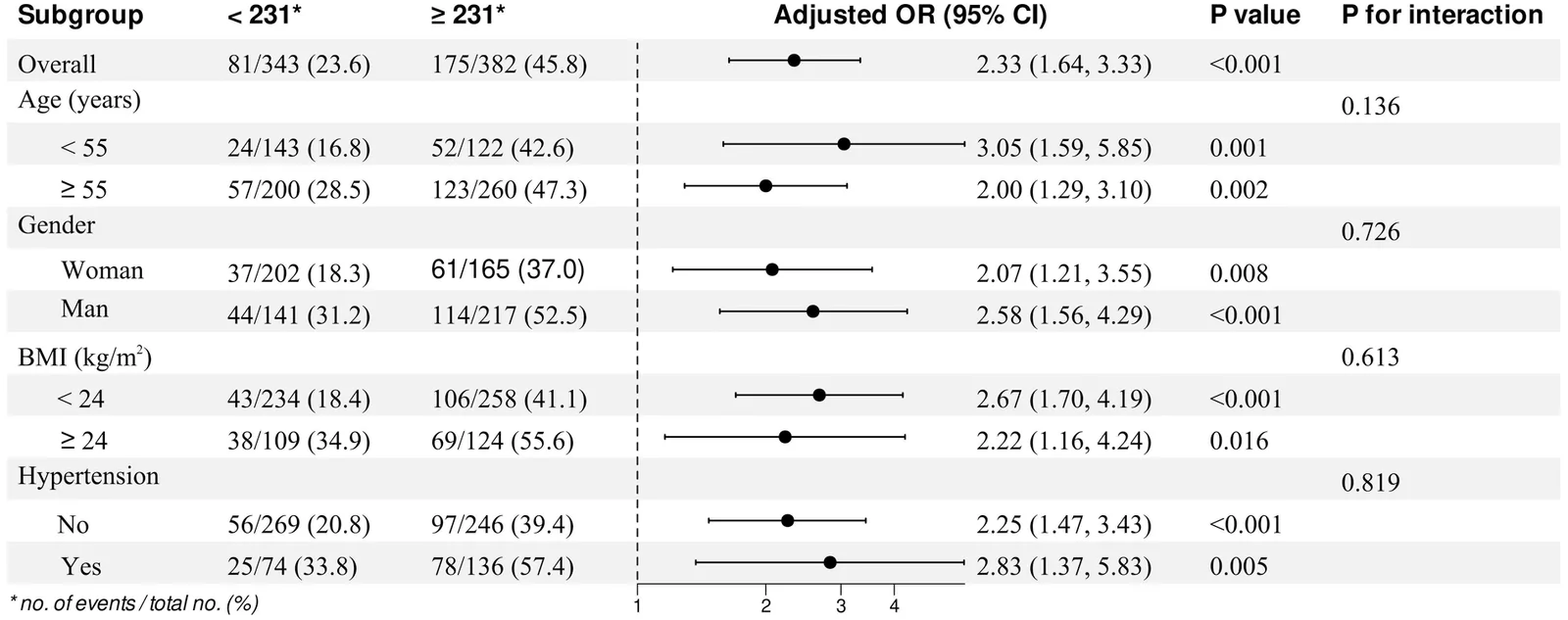
Subgroup analysis of the association between preoperative PIV and CSA-AKI. Forest plot showing the strength of the association between preoperative PIV and CSA-AKI across subgroups. The PIV cut-off of 231 was derived from the ROC analysis using the Youden index. Subgroup analyses were conducted based on age (<55 vs. ≥55 years), gender, body mass index (<24 vs. ≥24 kg/m^2^), and hypertension status. For each subgroup, the number of events/total patients (%) and the corresponding adjusted OR (95% CI) are presented; the dashed line denotes the overall effect. *P* for interaction assesses heterogeneity between subgroups. * PIV stratification: <231 vs. ≥231. ** Adjusted covariates include all variables listed in Table 2, Model 3.

### The incremental value of PIV

Incorporating PIV into the base model resulted in a continuous NRI of 24.82% (95% CI, 10.22%–39.41%; *p* < 0.001) and an IDI of 2.75% (95% CI, 1.39%–4.11%; *p* < 0.001), indicating significant enhancements in both discrimination and risk reclassification for CSA-AKI (Supplementary Table S8a). However, the categorical NRI was recorded at 2.42% (95% CI, −2.79% to 7.62%; *p* = 0.363), which did not achieve statistical significance, suggesting that PIV's contribution to clinically meaningful risk-category reclassification may be limited. The DCA demonstrated that both models conferred a greater net benefit than either the “treat all” or “treat none” strategy across threshold probabilities of approximately 10%–70% (Supplementary Figure S8b). Importantly, the PIV model achieved a higher net benefit than the base model at clinically relevant thresholds of 20%–65%, indicating that incorporation of PIV into the base model translates into a clinically meaningful improvement in decision-making.

### Mediation analysis

Given that intraoperative factors can mediate the association between preoperative PIV and CSA-AKI, these variables were intentionally excluded from the primary regression models. Notably, factors such as hypotension exposure, CPB duration, fluid administration, and blood transfusion volume are critical determinants of CSA-AKI risk and warrant consideration. Consequently, a mediation analysis was conducted to quantify the mediating roles of these intraoperative factors. As outlined in Supplementary Table S9, the analysis demonstrated that CPB duration (mediating 21.63% of the total effect) and intraoperative blood loss (14.95%) acted as significant mediators in the pathway linking elevated PIV to CSA-AKI. In contrast, exposure to intraoperative MAP < 60 mmHg, blood product transfusion, total fluid balance, and urine output were not identified as significant mediators. To further validate robustness, these non-significant mediators were included in a multivariable logistic regression model (Supplementary Table S10). In this adjusted model, preoperative PIV remained independently and positively associated with CSA-AKI (OR = 1.03, 95% CI: 1.01–1.06, *p* = 0.003), confirming the robust nature of the association between preoperative PIV and CSA-AKI, even after accounting for intraoperative variables.

## Discussion

The findings of our retrospective study indicate that patients who experienced CSA-AKI displayed significantly elevated preoperative PIV compared to those without CSA-AKI. After adjusting for confounding factors, PIV remained independently and monotonically associated with the incidence of CSA-AKI, with an attenuating slope at higher values. Consistency in the results of subgroup, mediation, and sensitivity analyses further strengthens the robustness of our conclusions. To our knowledge, studies specifically evaluating PIV in relation to CSA-AKI are limited.

Previous research has established that elevated NLR and SII independently predict CSA-AKI during CPB (20, 28). Our findings support these reports and extend them by illustrating that increased preoperative PIV is linearly and independently associated with CSA-AKI, highlighting its potential as an indicator of susceptibility to this condition. However, this study concentrated on the incidence of CSA-AKI in the early postoperative period. CSA-AKI is linked to a higher risk of CKD and long-term mortality (29). While earlier studies have explored how postoperative SII contributes to CSA-AKI (20), our investigation emphasizes the preoperative immune-inflammation-coagulation environment rather than the acute inflammatory surge caused by surgical trauma or CPB. By prioritizing preoperative PIV, this study proposes it as a potential risk-stratification tool that may guide renal-protective measures, fluid management strategies, or the use of anti-inflammatory adjuncts prior to surgery. Previous research indicated that preoperative SII values could predict postoperative AKI after coronary artery bypass grafting (30); our study builds on this by incorporating a more comprehensive PIV, allowing isolation of the pre-existing inflammatory load as an independent linear predictor. This approach broadens the prognostic focus from the post-CPB inflammatory response to the assessment of preoperative inflammatory burden. Future studies should investigate whether PIV can predict progression to CKD, significant declines in estimated glomerular filtration rate, or the need for dialysis (15).

In comparison to NLR and PLR, elevated SII levels may reflect increased inflammatory activity, resulting in renal tissue damage and heightened inflammation (31). PIV demonstrated a notably higher discriminative ability for CSA-AKI than NLR, PLR, SII, MLR, and MLPR. In addition to considering SII, PIV includes monocyte count, a key component of the inflammatory response. During the inflammatory cascade triggered by cardiac surgery, monocytes become activated, migrate to injured tissues, differentiate into macrophages, and modulate the inflammatory response (32). Consequently, PIV may more comprehensively capture the complexity of the inflammatory process, particularly the immunopathological aspects related to the monocyte/macrophage system. Nevertheless, the AUC for PIV remains suboptimal for clinical application, suggesting that PIV alone is unlikely to provide individualized, precise predictions. Moreover, the per-50-unit adjusted OR of 1.04 in the primary model represents a modest incremental effect size at the individual level, consistent with the discriminative capacity of PIV. However, this per-unit estimate should be interpreted in the context of PIV's wide distribution across the cohort; the quartile-based analysis provides a more clinically interpretable magnitude, with patients in the highest quartile (Q4 ≥ 427) exhibiting a 3.23-fold higher odds of CSA-AKI compared with those in the lowest quartile (Q1 < 150). This limitation likely arises from the multifactorial nature of CSA-AKI, which involves not only preoperative systemic inflammation but also intraoperative hemodynamic fluctuations, CPB-related perturbations, CPB time, albumin, N-terminal pro-B-type natriuretic peptide, and other critical determinants (33). It is proposed that PIV serves best as an adjunctive screening tool within a broader risk-assessment framework rather than as a standalone diagnostic criterion. Future research should integrate PIV into composite models that include dynamic intraoperative parameters to enhance predictive accuracy.

The systemic inflammatory response plays a critical role in initiating CSA-AKI by disrupting endothelial homeostasis, promoting renal ischemia-reperfusion injury, and exacerbating oxidative stress (16, 34, 35). Activated neutrophils release pro-inflammatory mediators and form neutrophil extracellular traps, which can directly damage renal tubular epithelial cells (36, 37). Lymphocytes and monocyte-derived macrophages contribute to the propagation of renal inflammation by increasing vascular permeability and modulating local immune responses (36, 38–40). Furthermore, platelet activation and coagulation dysfunction worsen these processes through microthrombus formation and reduced renal microvascular perfusion (41–44). Collectively, these immune-inflammatory and prothrombotic mechanisms provide a compelling biological basis for linking elevated preoperative PIV—an integrated marker of neutrophils, lymphocytes, monocytes, and platelets—to an increased risk of CSA-AKI.

Early identification of patients at high risk for CSA-AKI and optimizing CSA-AKI management strategies are vital for enhancing perioperative outcomes (45). Although renoprotective anti-inflammatory agents are not widely recommended, recognizing patients with elevated inflammatory risk based on preoperative PIV and implementing pharmacologic strategies to alleviate inflammation-related renal injury represent a plausible clinical approach. The preoperative PIV demonstrated incremental predictive value beyond established immune-inflammatory indices such as NLR, SII, MLR, and MLPR, yielding significant improvements in discrimination and overall reclassification. However, the categorical NRI was not statistically significant, indicating that PIV's capacity to shift patients across predefined clinical risk categories is limited. Consequently, while PIV may improve risk stratification as an easily accessible, preoperative immune-inflammatory marker, its incremental value should be interpreted with caution and complemented by clinical judgment and existing risk assessment tools.

## Limitations

Several limitations should be acknowledged. Despite adjustments made through multivariable regression and PSM, residual confounding from unmeasured variables remains a concern. PSM can only balance observed characteristics; thus, unmeasured biases may limit the generalizability of our causal inferences. CSA-AKI was analyzed as a binary outcome in this study. Owing to the limited sample size, we were unable to further explore the association between preoperative PIV and AKI severity (KDIGO stages). Therefore, the preoperative PIV is currently recommended only for predicting the occurrence of CSA-AKI; whether it can discriminate AKI severity warrants validation in larger cohorts with prospective creatinine surveillance. Additionally, the study did not evaluate serial postoperative PIV measurements or their relationship with AKI severity, renal recovery, or long-term outcomes. The incremental value of PIV must also be contextualized with the non-significant categorical NRI, as mentioned earlier.

## Conclusion

In summary, an elevated preoperative PIV is independently associated with an increased risk of CSA-AKI. Integrating preoperative PIV into risk-stratification tools could enhance the accuracy of identifying individuals at risk. Further studies are needed to validate these findings and thoroughly investigate the role of PIV in preoperative anti-inflammatory strategies for preventing CSA-AKI.

## Data Availability

The raw data supporting the conclusions of this article will be made available by the authors, without undue reservation.
