# Supplementary material for: Associations of preoperative pan-immune inflammation value with postoperative acute kidney injury following open-heart surgery: a retrospective analysis
Source: Front Cardiovasc Med. 2026 Sep 24;13:1920153. doi: 10.3389/fcvm.2026.1920153 (PMC13646072; doi:10.3389/fcvm.2026.1920153)
Supplement: Supplementary file 1 [file Datasheet1.pdf]

Supplementary materials

Supplementary Table S1 Different indices calculation formulas		
Indices	Formula	
NLR	Neutrophil-to-Lymphocyte Ratio	
SII	Platelet Count × Neutrophil Count / Lymphocyte Count	Systemic Immune-Inflammation Index
PLR	Platelet-to-Lymphocyte Ratio	
MLR	Monocyte-to-Lymphocyte Ratio	
MLPR	Monocyte-to-Lymphocyte × Platelet ratio.	

Supplementary Table S2 Missing information of variables		
	Missing information	
	Counts	Percentage (%)
Body mass index	10	1.4
Triglyceride-glucose index	82	11.3
Albumin	39	5.4
Serum creatinine	11	1.5
blood urea nitrogen	11	1.5
Uric acid	38	5.2
Hemoglobin	2	.3
Left ventricular ejection fraction	60	8.3
Blood output	12	1.7
Urine output	16	2.2
Crystalloid Volume	2	.3
The length of cardiopulmonary bypass	75	10.3
The length of aortic cross-clamping	79	10.9

Supplementary Table S3 Multicollinearity Diagnosis		
	Tolerance	Variance Inflation Factor
Preoperative PIV	0.683	1.465
Age	0.584	1.712
Gender	0.706	1.416
BMI	0.738	1.356
Hypertension	0.656	1.524
Diabetes	0.609	1.642
Ischemic heart diseases	0.752	1.330
Nicotine dependence	0.828	1.208
Albumin	0.815	1.227
TyG index	0.750	1.334
Serum creatinine	0.502	1.991
BUN	0.476	2.101
Uric acid	0.777	1.288
Hemoglobin	0.719	1.391
Antihypertensive medications	0.763	1.311
Lipid-lowering medications	0.723	1.384
Anti-diabetic medications	0.620	1.612
Diuretic medications	0.802	1.247
Preoperative LVEF	0.881	1.135
Coronary Angiography	0.740	1.352
ASA classifications	0.888	1.127
Surgical types	0.560	1.786
Blood output	0.628	1.591

Urine output	0.661	1.514
Crystalloid Volume	0.794	1.259
Colloid Volume	0.836	1.196
RBC transfusion	0.719	1.391
Plasma transfusion	0.743	1.346
The length of CPB	0.377	2.650
The length of ACC	0.205	4.871
TW-AUC MAP < 60 mmHg	0.875	1.143
The length of anesthesia	0.166	6.008

PIV: pan-immune inflammation value; BMI: body mass index; TyG: triglyceride-glucose index; BUN: blood urea nitrogen; LVEF: left ventricular ejection fraction. ASA classifications: American Society of Anesthesiologists; TW-AUC MAP <60 mmHg: the time-weighted area under the curve mean arterial pressure <60 mmHg; CPB: cardiopulmonary bypass; ACC: aortic cross-clamping; RBC: red blood cell.

Supplementary Table S4 Patient demographics and baseline characteristics

Characteristic	Preoperative PIV quartile range				
	Q1 (<150)<br>N = 181	Q2 (150 - 242)<br>N = 181	Q3 (243 - 426)<br>N = 181	Q4 (≥427)<br>N = 182	p
Preoperative PIV	101 (74, 128)	188 (168, 216)	316 (274, 359)	701 (551, 1,241)	<0.001
CSA-AKI	37 (20.4)	53 (29.3)	72 (39.8)	94 (51.7)	<0.001
Age (years)	58 (51, 64)	56 (49, 61)	60 (50, 65)	59 (52, 66)	0.003
Gender (woman)	120 (66.3)	88 (48.6)	84 (46.4)	75 (41.2)	<0.001
BMI (kg/m^2)	22.6 (19.9, 24.5)	22.7 (19.9, 24.8)	22.5 (20.4, 24.5)	22.3 (19.9, 25.2)	0.906
Preoperative medical histories					
Hypertension	41 (22.7)	39 (21.5)	55 (30.4)	75 (41.2)	<0.001
Diabetes	13 (7.2)	23 (12.7)	28 (15.5)	36 (19.8)	0.005
Ischemic heart diseases	91 (50.3)	88 (48.6)	105 (58.0)	92 (50.5)	0.283
Preoperative laboratory results					
Albumin (g/L)	40.0 (37.5, 41.8)	39.9 (37.3, 42.1)	39.0 (36.8, 41.9)	37.5 (34.7, 39.9)	<0.001
TyG index	7.09 (6.80, 7.47)	7.15 (6.81, 7.59)	7.14 (6.88, 7.51)	7.22 (6.90, 7.58)	0.228
Serum creatinine (umol/L)	78 (65, 92)	79 (68, 94)	87 (74, 102)	88 (73, 112)	<0.001
BUN (mmol/L)	5.70 (4.57, 7.05)	5.56 (4.61, 7.26)	6.57 (4.77, 8.10)	6.79 (4.93, 9.27)	<0.001
Uric acid (umol/L)	384 (326, 470)	401 (334, 496)	447 (367, 536)	409 (313, 503)	<0.001
Hemoglobin (g/L)	133 (124, 145)	136 (125, 149)	138 (125, 152)	132 (115, 144)	0.002
Preoperative medications					
Antihypertensive medications	113 (62.4)	118 (65.2)	130 (71.8)	145 (79.7)	0.002
Lipid-lowering medications	47 (26.0)	62 (34.3)	92 (50.8)	71 (39.0)	<0.001
Anti-diabetic medications	15 (8.3)	19 (10.5)	25 (13.8)	25 (13.7)	0.281
Diuretic medications	173 (95.6)	165 (91.2)	173 (95.6)	161 (88.5)	0.020
Preoperative LVEF (%)	63 (59,68)	63(57,67)	62(57,68)	62 (57,67)	0.646
Intraoperative information					
ASA classifications					<0.001

II-III	70 (38.7)	51 (28.2)	59 (32.6)	35 (19.2)	
IV-V	111 (61.3)	130 (71.8)	122 (67.4)	147 (80.8)	
Surgical types					<0.001
Great vessels	6 (3.3)	1 (0.6)	12 (6.6)	41 (22.5)	
Valves	139 (76.8)	129 (71.3)	127 (70.2)	110 (60.4)	
CABG and valves	4 (2.2)	7 (3.9)	10 (5.5)	7 (3.8)	
CABG	5 (2.8)	14 (7.7)	16 (8.8)	10 (5.5)	
Other surgeries	27 (14.9)	30 (16.6)	16 (8.8)	14 (7.7)	
Other preoperative indexes					
NLR	1.52 (1.25, 1.81)	2.02 (1.65, 2.39)	2.47 (2.10, 3.07)	4.40 (3.04, 6.89)	<0.001
SII	274 (216, 314)	418 (357, 501)	570 (486, 700)	1,083 (803, 1,664)	<0.001
PLR	92 (72, 112)	110 (90, 131)	123 (100, 154)	163 (126, 222)	<0.001
MLR	0.19 (0.15, 0.24)	0.23 (0.19, 0.28)	0.29 (0.25, 0.35)	0.50 (0.37, 0.69)	<0.001
MLPR	33 (26, 40)	49 (43, 57)	67 (57, 80)	113 (94, 158)	<0.001

The continuous variables are presented as median (interquartile range, 25%, 75%). The categorical variablesare expressed as counts (percentage). CSA-AKI: cardiac surgery associated-acute kidney injury; PIV: pan-immune inflammation value; BMI: body mass index;TyG: triglyceride glucose-body mass index; BUN: blood urea nitrogen; LVEF: left ventricular ejection fraction. ASA classifications: American Society of Anesthesiologists; CABG: coronary artery bypass grafting. Other surgery types included congenital heart disease surgery and cardiac myxoma surgery. SII: systemic immune-inflammation index; NLR: neutrophil-to-lymphocyte ratio; PLR: platelet-to-lymphocyte ratio; MLR: monocyte-to-lymphocyte ratio; MLPR: monocyte-to-lymphocyte × platelet ratio.

Supplementary Table S4 delineates the baseline characteristics of 725 patients stratified by preoperative pan-immune inflammation value (PIV) quartiles. The incidence of cardiac surgery-associated acute kidney injury (CSA-AKI) rose steadily across PIV quartiles, from 20.4 % in the lowest quartile ($PIV < 150$) to 51.7 % in the highest ($PIV \geq 427$) ($p < 0.001$). Patients with higher PIV were older, had a lower proportion of women, and exhibited greater rates of hypertension and diabetes (all $p < 0.05$). Preoperative laboratory profiles also differed significantly: albumin levels declined (40.0 g/L in Q1 vs. 37.5 g/L in Q4, $p < 0.001$), while serum creatinine, blood urea nitrogen, and uric acid

increased with rising PIV (all $p < 0.001$). Inflammatory indices—including systemic immune-inflammation index, neutrophil-to-lymphocyte ratio, monocyte-to-lymphocyte ratio, and monocyte-to-lymphocyte × platelet ratio—all climbed steeply across PIV quartiles (all $p < 0.001$). Collectively, the table demonstrates a graded, dose-dependent relationship between preoperative PIV and both CSA-AKI risk and a cluster of adverse preoperative and intraoperative characteristics.

Supplementary Table S5. Receiver Operating Characteristic Analysis of Preoperative Inflammatory Biomarkers for Predicting CSA-AKI			
	Preoperative Predictors	AUC	95% CI
CSA-AKI	PIV	0.641	0.599 - 0.683
	NLR	0.579	0.535 - 0.624
	SII	0.601	0.557 - 0.645
	PLR	0.486	0.441 - 0.531
	MLR	0.579	0.536 - 0.623
	MLPR	0.601	0.558 - 0.645
		P-value	
DeLong’s test was used to compare the predictive performance of preoperative PIV with that of the other predictors.	PIV vs. SII	<0.001	
	PIV vs. NLR	<0.001	
	PIV vs. PLR	<0.001	
	PIV vs. MLR	<0.001	
	PIV vs. MLPR	<0.001	

AUC: Area Under the Curve;CI: Confidence Interval;

CSA-AKI: cardiac surgery associated-acute kidney injury;

PIV: pan-immune inflammation value;

SII: systemic immune-inflammation index;

NLR: neutrophil-to-lymphocyte ratio;

PLR: platelet-to-lymphocyte ratio;

MLR:monocyte-to-lymphocyte ratio;

MLPR: monocyte-to-lymphocyte × platelet ratio.

Supplementary Table S6 Baseline characteristics before PSM and after PSM (patients stratified by PIV value 231)										
	Before PSM					After PSM				
	Total (n = 725)	<231 (n = 343)	≥231 (n = 382)	P	SMD	Total (n = 536)	<231 (n = 268)	≥231 (n = 268)	P	SMD
Age (years)	58 (50, 65)	57 (50, 62)	60 (51, 66)	0.003	0.207	58 (51, 65)	57 (51, 63)	59 (50, 65)	0.156	0.069
BMI (kg/m^2)	22.5 (20.0, 24.8)	22.6 (19.9, 24.7)	22. 5 (20.2, 24.9)	0.847	0.031	22.4 (20.0, 24.6)	22.6 (20.0, 24.7)	22.3 (20.1, 24.4)	0.287	-0.080
TyG	7.15 (6.82, 7.53)	7.11 (6.80, 7.52)	7.18 (6.88, 7.53)	0.083	0.135	7.13 (6.82, 7.53)	7.11 (6.81, 7.54)	7.14 (6.88, 7.51)	0.403	0.066
Albumin (g/L)	39.0 (36.6, 41.6)	39.9 (37.3, 41.9)	38.1 (36.2, 41.0)	<0.001	-0.287	39.1 (36.6, 41.7)	39.3 (36.7, 41.5)	38.7 (36.6, 42.0)	0.591	-0.018
Serum creatinine (umol/L)	81.9 (69.5, 100.7)	78.1 (65.4, 91.8)	86.8 (73.1, 104.6)	<0.001	0.293	81.0 (69.7, 97.8)	80.1 (68.3, 94.0)	82.7 (71.0, 99.5)	0.114	0.158
BUN (mmol/L)	6.08 (4.71, 7.89)	5.60 (4.58, 7.12)	6.76 (4.92, 8.71)	<0.001	0.359	6.01 (4.64, 7.56)	5.80 (4.76, 7.21)	6.21 (4.52, 7.92)	0.309	0.153
Uric acid (umol/L)	411 (335, 504)	392 (328, 486)	430 (344, 522)	<0.001	0.259	416 (335, 504)	416 (337, 508)	416 (333, 500)	0.804	0.046
Gender (woman)	367 (50.6)	202 (58.9)	165 (43.2)	<0.001	-0.317	273 (50.93)	140 (52.24)	133 (49.63)	0.545	-0.052
Hypertension	210 (29.0)	74 (21.6)	136 (35.6)	<0.001	0.293	142 (26.5)	66 (24.6)	76 (28.4)	0.328	0.083
Diabetes	100 (13.8)	32 (9.3)	68 (17.8)	<0.001	0.221	77 (14.4)	32 (11.9)	45 (16.8)	0.109	0.130
Ischemic heart diseases	376 (51.9)	170 (49.6)	206 (53.9)	0.240	0.088	294 (54.9)	148 (55.2)	146 (54.5)	0.862	-0.015
Antihypertensive medications	506 (69.8)	219 (63.9)	287 (75.1)	<0.001	0.261	374 (69.8)	186 (69.4)	188 (70.2)	0.851	0.016
Lipid-lowering medications	272 (37.5)	102 (29.7)	170 (44.5)	<0.001	0.297	205 (38.3)	100 (37.3)	105 (39.2)	0.657	0.038
anti-diabetic medications	84 (11.6)	31 (9.0)	53 (13.9)	0.042	0.140	70 (13.1)	30 (11.2)	40 (14.9)	0.200	0.105
Surgical types				<0.001					0.998	
CABG and valves	28 (3.7)	10 (2.9)	18 (4.7)		0.085	21 (3.9)	10 (3.7)	11 (4.1)		0.019
CABG	45 (6.2)	17 (5.0)	28 (7.3)		0.091	34 (6.3)	17 (6.3)	17 (6.3)		0.000
Others	87 (12)	54 (15.7)	33 (8.6)		-0.253	60 (11.2)	31 (11.6)	29 (10.8)		-0.024
Valves	508 (70.1)	256 (74.6)	252 (66.0)		-0.183	409 (76.3)	204 (76.1)	205 (76.5)		0.009
Great vessels	57 (7.9)	6 (1.8)	51 (13.4)		0.341	12 (2.2)	6 (2.2)	6 (2.2)		0.000
ASA classifications				0.094					0.925	

II-III	215 (29.7)	112 (32.7)	103 (27.0)		-0.128	163 (30.4)	81 (30.2)	82 (30.6)		0.008
IV-V	510 (70.3)	231 (67.4)	279 (73.0)		0.128	373 (69.6)	187 (69.8)	186 (69.4)		-0.008

The continuous variables are presented as median (interquartile range, 25%, 75%). The categorical variables are expressed as counts (percentage). PSM: propensity score matching; SMD: standardized mean difference; CSA-AKI: cardiac surgery associated-acute kidney injury; PIV: pan-immune inflammation value; BMI: body mass index; TyG: triglyceride-glucose index; TyG = ln[triglyceride(mg/dL) × fasting blood glucose (mg/dL)/2]; BUN: blood urea nitrogen; LVEF: left ventricular ejection fraction. ASA classifications: American Society of Anesthesiologists; CABG: coronary artery bypass grafting. Other surgery types included congenital heart disease surgery and cardiac myxoma surgery.

Supplementary Table S7 Logistic regression of PIV and CSA-AKI after PSM

Variables	β	S.E	Z	P	OR (95%CI)
PIV groups					
<231					1.00 (Reference)
≥231	0.65	0.18	3.52	<0.001	1.92 (1.33 ~ 2.76)

OR: Odds Ratio, CI: Confidence Interval, CSA-AKI: cardiac surgery associated-acute kidney injury; PIV: pan-immune inflammation value; PSM: propensity score matching;

Supplementary Table S8a Comparing the Predictive Performance of the PIV and the Base Model		
Indicators	Estimate (95% CI)	P-value
NRI（Categorical）	0.0242 (-0.0279, 0.0762)	0.363
NRI（Continuous）	0.2482 (0.1022, 0.3941)	<0.001
IDI	0.0275 (0.0139, 0.0411)	<0.001

Abbreviations: NRI, net reclassification improvement; IDI, integrated discrimination improvement. The PIV model is an extended model incorporating the PIV index. The base model served as the reference and excluded the PIV index. Variables for the base model were selected via LASSO regression and included age, preoperative albumin, preoperative triglyceride-glucose (TyG) index, preoperative creatinine, and type of surgery.

Supplementary Figure S8b

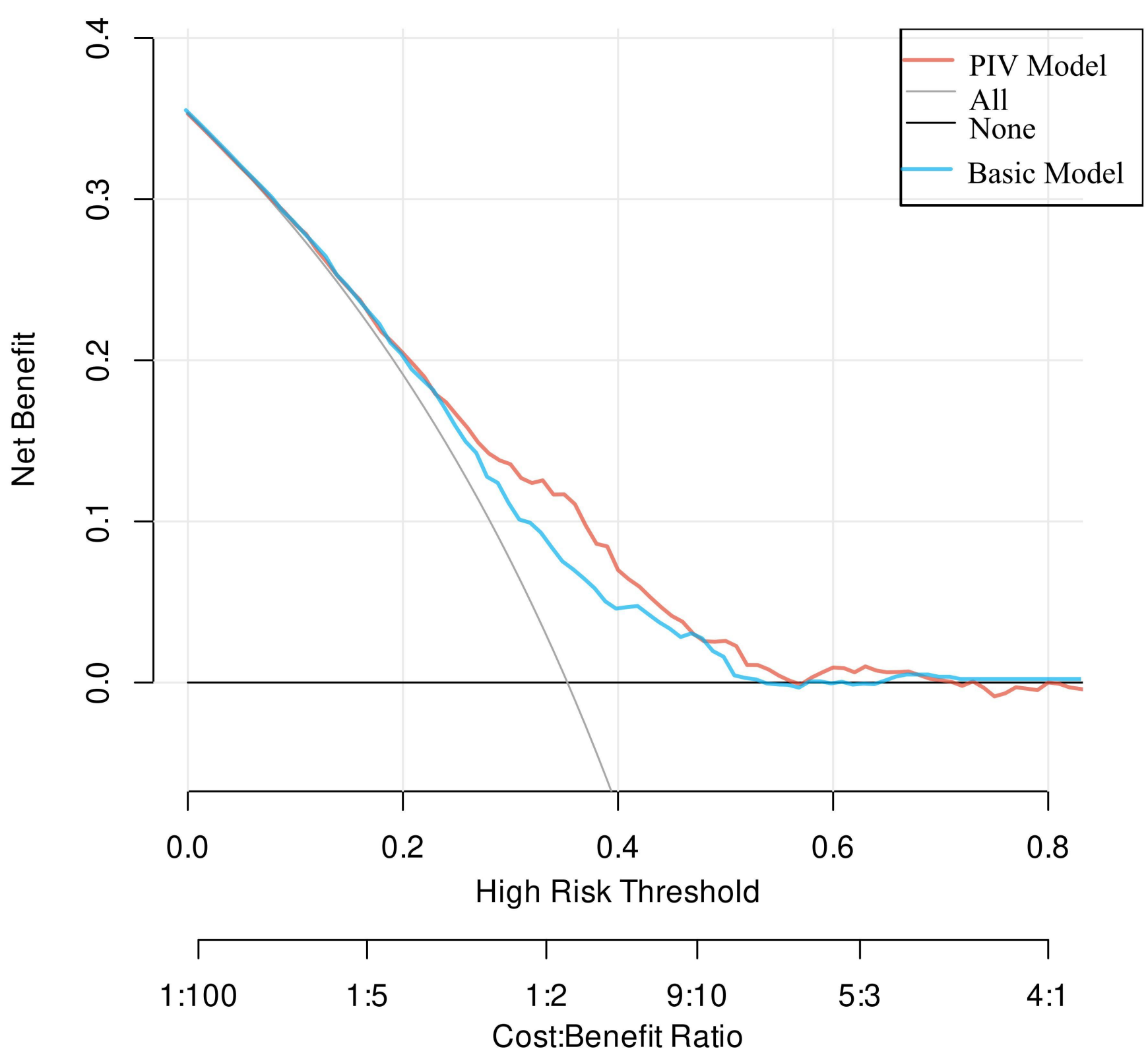

Decision curve analysis demonstrated that both models yielded greater net benefit than the “treat all” or “treat none” strategies across threshold probabilities of approximately 10%–70%. Notably, the PIV model provided a higher net benefit than the base model within the clinically relevant threshold range of 20%–65%, with the largest gain observed at approximately 35%—corresponding closely to the observed CSA-AKI incidence (35.3%)—indicating that incorporating PIV into the base model translates into a meaningful improvement in clinical decision-making.

Supplementary Table S9. Mediation analysis results for the association between preoperative PIV and CSA-AKI							
Mediator	a path (X→M)	b path (M→Y)	Total effect (c path)	Direct effect (c' path)	Indirect effect (a×b)	P value (indirect)	Proportion mediated (%)
	β (P)	OR (P)	OR (P)	OR (P)	β (95% CI)		
CPB duration	0.02 (<0.001)	1.01 (<0.001)	1.01 (<0.001)	1.01 (0.002)	0.0 (0.00 ~ 0.00)	<0.001	21.63
MAP <60 mmHg	0.01 (<0.001)	1.01 (0.723)	1.01 (<0.001)	1.01 (<0.001)	0.0 (-0.00 ~ 0.00)	0.760	1.42
RBC transfusion	0.01 (<0.001)	2.5 (<0.001)	1.01 (<0.001)	1.01 (0.001)	-0.0 (-0.01 ~ 0.01)	0.880	—
Blood loss	0.1 (<0.001)	1.0 (0.060)	1.01 (<0.001)	1.01 (0.001)	0.0 (0.00 ~ 0.00)	<0.001	14.95
Urine output	-0.05 (0.313)	0.99 (0.027)	1.01 (<0.001)	1.01 (<0.001)	0.0 (-0.00 ~ 0.00)	0.320	1.46
Crystalloid volume	0.02 (0.457)	0.99 (0.040)	1.01 (<0.001)	1.01 (<0.001)	-0.0 (-0.00 ~ 0.00)	0.440	-1.24
Plasma transfusion	0.01 (<0.001)	1.36 (0.080)	1.01 (<0.001)	1.01 (<0.001)	-0.0 (-0.00 ~ 0.00)	0.880	—

PIV, pan-immune inflammation value; CSAAKI, cardiac surgery-associated acute kidney injury; CPB, cardiopulmonary bypass; MAP, mean arterial pressure; RBC, red blood cell; OR, odds ratio; β, regression coefficient; CI, confidence interval.

a path: effect of PIV on mediator (linear regression β for continuous mediators; logistic regression β for categorical mediators).

b path: effect of mediator on CSAAKI controlling for PIV (logistic regression OR). For categorical mediators (RBC, Plasma), OR represents M=1 vs M=0.

c path: total effect of PIV on CSAAKI (logistic regression, without mediator); c' path: direct effect of PIV on CSAAKI (logistic regression, controlling for mediator).

Indirect effect (a×b) and 95% CI were estimated using bootstrap method with 5000 resamples. Proportion mediated = indirect effect / total effect × 100%.

Gray text indicates non-significant indirect effects (P ≥ 0.05 or 95% CI includes 0). "—" indicates proportion mediated not interpretable due to non-significant total or indirect effect.

All models adjusted for: sex, hypertension, diabetes, coronary artery disease, antihypertensive drugs, lipid-lowering drugs, oral hypoglycemic drugs, ASA classification, age, BMI, TyG index, preoperative albumin, creatinine (eGFR), urea nitrogen, uric acid.

Results Description

We conducted mediation analyses to evaluate whether the association between preoperative PIV and CSA-AKI was mediated by seven candidate intraoperative variables: cardiopulmonary bypass (CPB) duration, duration of intraoperative mean arterial pressure (MAP) <60 mmHg, red blood cell (RBC) transfusion, intraoperative blood loss, urine output, crystalloid volume, and plasma transfusion. All models were adjusted for sex, hypertension, diabetes, coronary artery disease, use of antihypertensive, lipid-lowering, and oral hypoglycemic agents, ASA classification, age, body mass index (BMI), triglyceride-glucose (TyG) index, preoperative albumin, estimated glomerular filtration rate, blood urea nitrogen, and uric acid. Preoperative PIV demonstrated a significant total effect on CSA-AKI across all models (c-path: OR = 1.01, 95% CI: 1.01–1.06, P < .001). CPB duration and intraoperative blood loss were identified as significant mediators, accounting for 21.63% and 14.95% of the total effect, respectively. The remaining five variables—duration of MAP <60 mmHg, RBC transfusion, plasma transfusion,

crystalloid volume, and urine output—did not exert significant mediating effects. These findings indicate that CPB duration and intraoperative blood loss are the primary pathways through which elevated PIV contributes to CSA-AKI risk, whereas hemodynamic instability, blood product transfusion, fluid management, and urine output do not serve as significant mediators in this association.

Supplementary Table S10. Multivariable Logistic Regression Analysis of Preoperative PIV and CSA-AKI Risk (included intraoperative variables)

Variables	Events（%）	Model	
		OR (95%CI)	P
Continuous PIV per 50 units	256 (35.3)	1.03 (1.01 ~ 1.06)	0.003
PIV quartile groups			
Q1 (<150)	37 (20.4)	Reference	Reference
Q2 (150 - 242)	53 (29.3)	1.31 (0.77 ~ 2.24)	0.326
Q3 (243 - 426)	72 (39.8)	2.03 (1.20 ~ 3.43)	0.009
Q4 (≥427)	94 (51.7)	2.67 (1.55 ~ 4.61)	<0.001

Adjust for: age, gender, BMI, hypertension, diabetes, ischemic heart disease, preoperative albumin, TyG index, creatinine, BUN, uric acid, antihypertensive agents, lipid-lowering drugs, and anti-diabetic drugs, ASA class, TW-MAP <60 mmHg, crystalloid volume, RBC transfusion, plasma transfusion, urine output, and surgical category.

Abbreviations: OR: Odds Ratio, CI: Confidence Interval; CSA-AKI: cardiac surgery associated-acute kidney injury; PIV: pan-immune inflammation value; BMI: body mass index; TyG: triglyceride-glucose index; BUN: blood urea nitrogen; ASA classifications: American Society of Anesthesiologists; TW-AUC MAP <60 mmHg: the time-weighted area under the curve mean arterial pressure <60 mmHg; RBC: red blood cell.
